# Comparison of short term results of single injection of autologous blood and steroid injection in tennis elbow: a prospective study

**DOI:** 10.1186/1749-799X-8-10

**Published:** 2013-04-27

**Authors:** Nipun Jindal, Yusuf Gaury, Ramesh C Banshiwal, Ravinder Lamoria, Vikas Bachhal

**Affiliations:** 1 Government Medical College and Hospital, Chandigarh, India; 2 Medical and Health Department, Jaipur, Rajasthan, India; 3 Sawai Man Singh Medical College and Hospital, Jaipur, Rajasthan, India; 4 Post Graduate Institute of Medical Education and Research, Chandigarh, India

**Keywords:** Tennis Elbow, Steroid Injection, Autologous Blood Injection

## Abstract

**Background:**

It has been recently reported that local injection of autologous blood in tennis elbow offers a significant benefit by virtue of various growth factors contained therein. The objective of our study was assessment of efficacy of autologous blood injection versus local corticosteroid injection in the treatment of tennis elbow.

**Methods and trial design:**

A single blinded, prospective parallel group trial was undertaken. 50 consecutive patients of untreated lateral epicondylitis were enrolled. Randomisation was done on alternate basis and two groups were constituted, first one receiving steroid injection and second one injection of autologous blood. Both groups were evaluated at 2 and 6 weeks for pain relief and stage of disease.

**Results:**

Baseline evaluation showed no difference between the two groups (chi square test, P > 0.05). Between group analysis at 2 weeks showed no difference in pain relief and Nirschl stage (unpaired *t* test, P > 0.05). Evaluation at 6 weeks demonstrated a significant decrease in pain levels and stage of disease in blood group (unpaired *t* test, p < 0.05).

**Conclusions:**

Autologous blood injection was more effective than steroid injection in the short term follow up in tennis elbow.

## Background

Lateral tendinosis of the elbow popularly known as tennis elbow refers to a degenerative process in the common origin of the extensor group of muscles of the forearm. The disorder arising as a result of repetitive movements of the involved muscles is a common cause of elbow pain particularly in the working age group. The disease imparts significant disability to those affected in terms of the quantity and quality of work done. Tennis elbow may cause significant weakness of grip strength particularly with the elbow in extension affecting a vast majority of daily life activities [1].

Although the diagnosis of lateral tendinosis is quite straight forward, there has been no consensus on the optimal management strategy [2]. Different treatment approaches exist with numerous and sometimes contradictory options with different mechanisms of action. With reports of tennis elbow being a degenerative process rather than an inflammatory one [3], the entire plethora of modalities aimed at arresting the inflammatory cascade seem ineffective. Nevertheless local injection of steroid has been proven to impart a consistent and predictable good short term pain relief [4]. Delivery of autologous blood derived growth factors to the site of disease has also been shown to significantly help the healing process in tennis elbow [5]. These growth factors can be delivered by an injection of whole blood or platelet concentrate. However scientific clinical evidence supporting incorporation of such modalities into routine clinical practice is scanty at present [6,7].

This trial was thus undertaken in patients of tennis elbow, to compare the short term benefits of a single injection of steroid and autologous blood in terms of pain relief and downstaging of the disease.

## Methods

### Trial design

This study was single blinded, parallel group trial involving untreated patients of lateral tendinosis of the elbow joint reporting to a tertiary level hospital from August 2009 to August 2010 with an equal allocation ratio (1:1) to both groups. The study was approved by Ethics Committee of SMS Medical College and attached Hospitals with approval number 32167. The trial was registered with the institutional review board only in the absence of a nationwide trial registry in India at the time the study was instituted.

### Participants

Eligible participants were all the patients who were previously untreated and had no other identifiable cause of lateral elbow pain. A detailed clinical history was taken in all patients particularly regarding the degree of pain and the activity restrictions. Standard anteroposterior and lateral views of the elbow were obtained in all cases to rule out other causes of lateral elbow pain like radiocapitellar arthritis. Those reporting with the typical symptoms of tennis elbow and having no radiographic cause of pain were included in our study.

All the patients gave informed consent to participate in the study and were given the right to withdraw from the study at any time without any reprisal. 50 cases were selected to participate in our study.

### Interventions

Two groups were formed with one group receiving local steroid injection and the other one local injection of autologous blood. The cases were allotted to the groups on alternate basis. In Group I, 40 mg of methyl prednisolone acetate was used along with 1ml of 2% lignocaine solution. In Group II, 2 ml of venous blood was drawn from the ipsilateral or the contralateral upper limb and was injected after mixing with 1 ml of 2% lignocaine solution. The injection was administered in the outpatient department itself observing all aseptic precautions by same author (YG) in all the cases. The needle was introduced just proximal to the lateral epicondyle and the contents were injected on the undersurface of the extensor carpi radialis group of muscles. Patients were advised to restrain from activities involving repetitive movements of the wrist and elbow during initial 3 weeks after injection. Gentle passive stretching exercises of the extensor group of muscles was started as soon as the pain permitted.

### Outcome

The evaluation of the patients was carried out by the degree of the pain and the amount of disability in the pre injection phase, and at subsequent outpatient visits at 2 and 6 weeks. The degree of pain was assessed by employing the Visual Analogue scale (VAS) and the degree of disability was evaluated by Nirschl staging [8]. Outcome assessment was conducted by an independent observer (RL) blinded to the type of intervention received by an individual.

### Sample size

The minimum sample size required for this study was calculated on the basis of VAS pain intensity measurements. A significance criterion of 0.05 and power of 90% was chosen. Minimum expected difference between the two groups was chosen to be 1.5 on the pain scale. The minimum sample size came out to be 21 for each group based on the formula described for comparative research studies by Eng [9]. Standard deviation was taken to be 1.5 based on the values provided by Edwards et al. Based on an anticipated dropout rate of 10%, 50 patients were enrolled with 25 in each group. A post hoc analysis revealed that the study achieved a power of 84.7%.

### Statistical analysis

Chi square test was employed in comparison of baseline pattern of two groups. Paired *t* test was used for serial analysis in both groups and unpaired *t* test for comparison between the two groups. Statistical analysis was done using SPSS v.19 software (SPSS Inc., Chicago, Illinois).

## Results

A total of 50 patients with untreated lateral elbow tendinopathy of varying duration were included in this study during the time period from August 2009 to August 2010. The two groups comprised of 25 patients each, quasi randomized by alternate allocation. All the patients were administered treatment and analyzed as per treatment protocol.

### Baseline characteristics

A comparison of the baseline demographic and clinical data in both the groups was done (Table 1). Upon application of statistical tests, the difference between the two groups was found out to be non significant.

**Table 1:** Baseline clinical and demographic characteristics of each group

	**Group I (n=25)**	**Group II (n=25)**	**p value**
Age^#^(in years)	37.32 (7.52)	39.04 (6.67)	0.3965 (NS)
Sex*(males/females)	17/8	14/11	0.2268 (NS)
Laterality* (Right/Left)	21/4	23/2	0.1404 (NS)
Mean duration of symptoms# (in weeks)	4.4 (2.38)	4.48 (1.82)	0.8944 (NS)
Mean VAS Score#	6.2 (1.61)	5.88 (1.83)	0.5147 (NS)
Mean Nirschl stage #	4.84 (0.94)	4.52 (1.23)	0.3065 (NS)

*chi square test, ^#^unpaired *t* test, NS – not significant.

### Group I

The mean VAS scores in the pre injection phase was 6.20 ± 1.61. The pain decreased to a mean VAS of 3.52 ± 1.19 after 2 weeks of steroid injection. The mean VAS at 6 weeks of follow up was 2.28 ± 1.28. Similarly the mean value of Nirschl stage in Group 1 before administration of steroid was 4.84 ± 0.94. The mean Nirschl stage at 2 week and 6 week follow up was 3.20 ± 0.91 and 2.40 ± 1.15 respectively. The mean decrease observed in the VAS scores and Nirschl stage at 2 week and 6 week follow up after steroid injection came out to be highly significant (p<0.0001) (Table 2).

**Table 2:** Ealuation of outcome in steroid group

**Group I**	**Pre injection to 2 weeks FUP**	**Pre injection to 6 weeks FUP**
VAS [mean (SD)]	6.20 (1.61) to 3.52 (1.19)	6.20 (1.61) to 2.28 (1.28)
Statistical significance*	P < 0.0001 (HS)	P < 0.0001 (HS)
Nirschl Stage [mean (SD)]	4.84 (0.94) to 3.20 (0.91)	4.84 (0.94) to 2.40 (1.15)
Statistical significance*	P < 0.0001 (HS)	P < 0.0001 (HS)

*Paired *t* test, HS - highly significant.

### Group II

The baseline pain according to mean VAS score was 5.88 ± 1.83. The mean VAS score observed at 2 week and 6 week review came out to be 4.24 ± 1.64 and 1.52 ± 1.26 respectively. After application of paired *t* test, the p value for the fall in mean VAS score came out to be less than 0.0001 suggesting a extremely significant decrease in pain levels at both 2 and 6 week follow up. The mean Nirschl stage before blood injection was 4.52 ± 1.23 which subsequent to intervention decreased to 3.48 ± 1.39 at 2 weeks of follow up. The stage further decreased to a mean of 1.40 ± 1.22 at 6 week review. The change in Nirschl stage at both 2 and 6 weeks of review in Group 2 came out to be highly significant (p<0.0001) on paired *t* test (Table 3).

**Table 3:** Evaluation of outcome in blood group

**Group II**	**Pre injection to 2 weeks FUP**	**Pre injection to 6 weeks FUP**
VAS [mean (SD)]	5.88 (1.83) to 4.24 (1.64)	5.88 (1.83) to 1.52 (1.26)
Statistical significance*	P < 0.0001 (HS)	P < 0.0001 (HS)
Nirschl Stage [mean (SD)]	4.52 (1.23) to 3.48 (1.39)	4.52 (1.23) to 1.40 (1.22)
Statistical significance*	P < 0.0001 (HS)	P < 0.0001 (HS)

*Paired *t* test, HS - highly significant.

The above observations reveal that both steroid and blood injections independently gave a significant pain relief and significantly downstaged the disease in a short term follow up.

### Between group analysis

**Figure 1:**
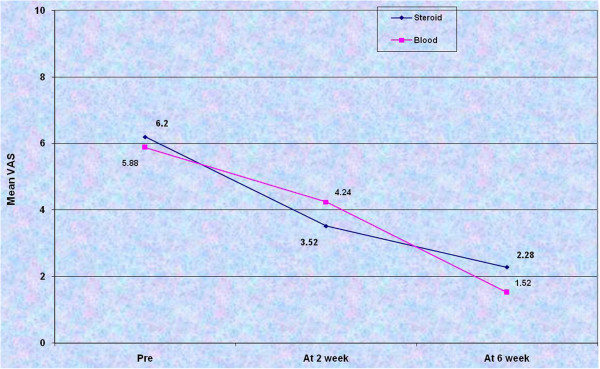
Line plot of the mean VAS scores in the pre injection phase, at 2 week follow up and at 6 weeks of review in both the groups.

After an initial analysis of the two groups separately, comparisons were drawn. The pre injection mean VAS score and the mean value of the Nirschl stage was similar in both groups. The mean VAS scores in both the groups were plotted on a line diagram (Figure 1).

Clinically it was found that pain relief at 2 weeks was more in steroid group while it was more at 6 weeks of review in blood group. However, statistical comparison between the two groups revealed that the mean VAS values at 2 weeks in both groups were similar while at 6 weeks it was significantly lower in group II (Table 4).

**Table 4:** Between-group comparison of Mean VAS scores at baseline and after 2 and 6 weeks of treatment

	**Pre injection**	**At 2 weeks**	**At 6 weeks**
Group I	6.20 (1.61)	3.52 (1.19)	2.28 (1.28)
Group II	5.88 (1.83)	4.24 (1.64)	1.52 (1.26)
P value#	0.5147	0.0820	0.0396
Significance	NS	NS	Significant

^#^unpaired *t* test, NS – not significant.

**Figure 2:**
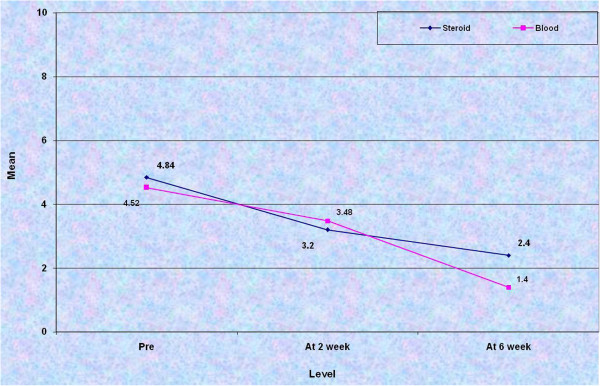
Line diagram depicting the mean Nirschl stage in pre-injection phase, at 2 weeks and 6 weeks post-injection in both the groups.

Similar to the above comparison, Nirschl stage was compared at 2 and 6 weeks post injection in both the groups (Figure 2). At 2 weeks, although the downstaging in group I was clinically better, statistical analysis yielded no significant result between the two groups. The comparison of mean VAS and mean Nirschl stage at 6 weeks of review came out to be highly significant indicating superiority of blood over steroid (Table 5).

**Table 5:** Between-group comparison of Mean Nirschl stage at baseline and after 2 and 6 weeks of treatment

	**Pre injection**	**At 2 weeks**	**At 6 weeks**
Group I	4.84 (0.94)	3.20 (0.91)	2.40 (1.15)
Group II	4.52 (1.23)	3.48 (1.39)	1.40 (1.22)
P value #	0.3065	0.436	0.0045
Significance	NS	NS	HS

^#^unpaired *t* test, NS – not significant, HS – highly significant.

The relief of pain from pre injection phase to 6 weeks review was graded as fair (0–3), good (4–6), excellent (≥7). The relief was excellent in 3 (12%) and 1 (4%) patients in blood and steroid groups respectively. Good and fair results were obtained in 14 (56%) and in 8 (32%) patients in blood group and 12 (48%) and 12 (48%) patients correspondingly.

## Discussion

Lateral elbow epicondylopathy remains one of the most perplexing disorders of the musculoskeletal system. It was first described by Runge [10] in 1873. The designation lateral epicondylitis is a misnomer since it has been proved that it is primarily a disorder related to degeneration in the tendon of common extensor origin (mostly extensor carpi radialis brevis) rather than inflammatory process as was thought to be earlier [3]. Maffuli et al. [11] recognised that tendinopathy is a clinical diagnosis while tendinitis or tendinosis terms should be reserved only after histopathological examination has been carried out. A cadaveric study by Bales et al. [12] showed two zones of hypovascularity in the region of lateral epicondyle, one between the lateral epicondyle and the supracondylar ridge and the other on the deep surface of common extensor tendon 2–3 cms distal to lateral epicondyle. This is probably the area where the degenerative changes set in (probably the same way as the supraspinatus tendinitis arises in a hypovascular zone just proximal to tendon insertion). The term Tennis elbow is not too apt since this disorder is commoner in occupations which involve repetitive forearm rotational activities. It has been estimated that only 5-10% of cases occur in tennis players [13]. In our series, 52% cases were manual workers most of them mechanics, 24% were homemakers mostly females and rest 24% were involved in sedentary life style with teachers constituting a substantial 8%.

The optimal treatment strategy for lateral elbow tendinopathy has baffled treating doctors for long. A large percentage of cases (70-80%) report resolution of their symptoms within a year with or without treatment [4]. Conservative line of management is usually the first line of treatment and consists of activity modification, rest, RICE (Rest, Ice, compression, elevation) therapy and non steroidal anti-inflammatory drugs [14]. Manipulation under anesthesia has been reported to yield good results [15]. Local corticosteroid injection is one of the commonest treatment prescribed in cases where initial activity modification and NSAIDs don’t work. However, a randomised control trial conducted by Bisset et al. [16] found out that corticosteroid although effective at short term yielded poorer results at long term follow up (1 year) than physiotherapy.

Several nonsurgical modalities of treatment are under investigation which upon preliminary research have reported to provide some relief in symptoms of tennis elbow. Plazcek et al. [17] investigated the role of Botulinum A toxin injection and found out that it offered significant pain relief. They reported significant weakness in the extensor movement of third finger but none of the patients lost time from work due to this weakness. D’Vaz [18] conducted a double blinded randomised controlled trial and concluded that pulsed low intensity ultrasound therapy offered no significant benefit over placebo.

Recent reports have emerged suggesting a beneficial role of growth factors delivered locally at the site of tendinopathy. This can be accomplished by injection of autologous blood or platelet concentrates. Mishra and colleagues [19] conducted a study wherein they treated patients of lateral elbow tendinopathy of less than 6 weeks duration by local injection of platelet rich plasma. They reported a significant improvement in pain. Similarly Edwards et al. [5] reported dramatic relief in symptoms in 28 patients of tennis elbow after injection of autologous blood. They postulated that autologous blood initiated an inflammatory reaction which allowed healing in otherwise degenerative process. Although this study didn’t involve any control group, the authors hypothesized that blood injection would provide additional benefits over an injection of either saline or steroid. This belief was based on their observations that blood injections provided relief to patients who had failed multiple steroid injection attempts despite similar injection techniques and volumes. A systematic review done by Vos et al. [20] however found that autologous blood has limited application in the management of tendinopathy. This was concluded on the basis of three studies [21-23] which involved management of plantar fasciitis with injection of autologous blood. The desired results may have not been achieved since the mechanical and healing properties of weight bearing and non weight bearing tendons differ a lot.

In our study, comparison between the two groups showed that both pain values and stage of the disease were similar at 2 weeks of review but there was a significant difference in both values at 6 weeks of follow up. Statistical analysis concluded that autologous blood was better than local corticosteroid injection in short term follow up of tennis elbow patients. This result came in direct consistency with study of Edwards et al. [5] who reported maximal pain relief 3 weeks after injection of autologous blood (clinically pain relief was better at 2 weeks in steroid group in our study). Kazemi et al. [24] also reported in their trial, that the benefits afforded by autologous blood injection outweighed those by local corticosteroid injection.

The mechanism of action of both autologous blood and platelet rich plasma is attributed to degranulation of α granules of platelets releasing growth factors which play a role in tissue healing and regeneration. Platelet derived growth factor, transforming growth factor β, vascular derived endothelial growth factor, epithelial growth factor, hepatocyte growth factor and insulin like growth factor are some of the factors involved [25]. Platelet rich plasma (PRP) varies in the relative concentration of platelets ranging from 2.5 to 5 times as compared to blood. PRP logically appears to be more effective than due to higher concentration of growth factors per unit volume and has been proved in one clinical trial to be better than autologous blood. However preparation of platelet concentrates requires specialised equipment which is both expensive and time consuming. Autologous blood has a far easier and prompt application than PRP.

The mechanism of action of steroid remains obscure. Balasubramaniam et al. [27] theorized that the beneficial effects of steroid injection result from the bleeding caused by forcing fluid through tissue planes at high pressures. In a study by Wolf et al. [28] corticosteroid, autologous blood and saline injection all afforded the same benefit in cases of tennis elbow. This indirectly points out that these reported outcomes may also be due to the placebo effect of injection itself or a reflection of concurrent resolution of a self-limited disease. One might be led to believe that the disparity in the efficacy of autologous blood and corticosteroid observed in some series is due to the relative difference in the quantity of growth factors delivered to the degenerated tendon. However this doesn’t explain the fact that maximal relief encountered by both modalities peaks at different times.

We acknowledge that the major limitation of our study is a shorter follow up. Long term follow up study is required to test the ability of blood injection to maintain its analgesic effect for a longer time. However at longer follow up the contribution of treatment to relief begins to dilute as the natural tendency of the disease to heal starts manifesting in the form of pain relief. Further studies are required to optimise the number and spacing of injections for obtaining desired results. Short duration of symptoms in our series might be perceived as a potential limitation but since the threshold for steroid injection in tennis elbow is low, many of the patients with a longer duration of symptoms had invariably been treated with a steroid injection elsewhere and thus couldn’t be included in the study. As a corollary, since we enrolled only those patients which were previously untreated, further studies are needed to detect any difference in efficacy of blood injection in cases earlier treated with other modalities. Further research can also be directed at elucidating the exact mechanism of action of blood and steroid, which can be known only by indirect methods in human subjects.

## Conclusion

Local injection of autologous blood offers a significant benefit over single steroid injection in the short term review in the treatment of patients of lateral elbow tendinopathy.

### Consent

Written informed consent was obtained from all the patients for publication of this study.

## Competing interests

No competing interests exist for this manuscript.

## Authors’ contributions

NJ drafted the manuscript and carried out the statistical analysis. YG administered the injections and carried out the baseline evaluation. RCB supervised the procedure and conceived the study. RL was blinded to the groups and reviewed the patients after the intervention. VB helped in literature review. All authors read and approved the final manuscript.

## Authors’ information

Study Conducted in: Sawai Man Singh Medical College and Hospital, Jaipur, Rajasthan, India
